# Nanostructured Niobium and Titanium Carbonitrides as Electrocatalyst Supports

**DOI:** 10.1021/acsanm.4c00503

**Published:** 2024-04-24

**Authors:** Lucy K. McLeod, Geoffrey H. Spikes, Christopher M. Zalitis, Katie M. Rigg, Marc Walker, Helen Y. Playford, Jonathan D. B. Sharman, Richard I. Walton

**Affiliations:** † Department of Chemistry, University of Warwick, Gibbet Hill Road, Coventry CV4 7AL, U.K.; ‡ Johnson Matthey Technology Centre, Blounts Court, Sonning Common, Reading RG4 9NH, U.K.; § Department of Physics, University of Warwick, Gibbet Hill Road, Coventry CV4 7AL, U.K.; ∥ ISIS Neutron and Muon Source, Rutherford Appleton Laboratory, Didcot OX11 0QX, U.K.

**Keywords:** carbide, oxygen evolution reaction, iridium, water splitting, electrocatalysis

## Abstract

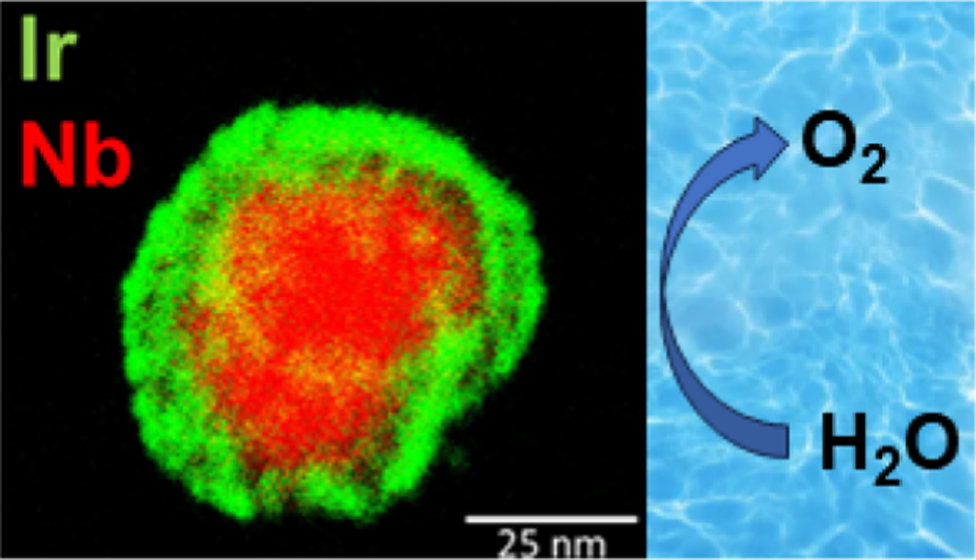

Nanostructured niobium–titanium carbonitrides, (Nb,Ti)C_1–*x*_N_*x*_, with the cubic-rock salt structure are prepared without the use of reactive gases via thermal treatment (700–1200 °C) under nitrogen of mixtures of guanidine carbonate and ammonium niobate (V) oxalate hydrate, with addition of ammonium titanyl oxalate monohydrate as a titanium source. The bulk structure and chemical composition of the materials are characterized using powder X-ray diffraction (XRD) and powder neutron diffraction, elemental homogeneity is studied using energy dispersive spectroscopy (EDS) mapping using transmission electron microscopy (TEM), and surface chemical analysis is examined using X-ray photoelectron spectroscopy (XPS). Nanoscale crystallites of between 10 and 50 nm are observed by TEM, where EDS reveals the homogeneity of metal distribution for the mixed-metal materials. Titanium carbonitrides are found to be air sensitive, reacting with air under ambient conditions, while titanium–niobium carbonitrides are found to degrade in aqueous sulfuric acid. The niobium carbonitrides, however, show some stability toward acidic solutions. Materials are produced with composition NbC_1–*x*_N_*x*_ with *x* between 0.35 and 0.45, and more carbon-rich materials (*x* ≈ 0.35) are found as the synthesis temperature is increased, as proven by Rietveld refinement of crystal structure against powder neutron diffraction data. Despite phase purity seen by diffraction and negligible bulk carbon content, XPS shows a complex surface chemistry for the NbC_1–*x*_N_*x*_ materials, with evidence for Nb_2_O_5_-like oxide species in a carbon-rich environment. The NbC_1–*x*_N_*x*_ prepared at 900 °C has a surface area around 50 m^2^ g^–1^, making it suitable as a catalyst support. Loading with iridium provides a material active for the oxygen evolution reaction in 0.1 M sulfuric acid, with minimal leaching of either Nb or Ir after 1000 cycles.

## Introduction

Carbides of transition metals have a variety of properties suitable for practical applications, including their electronic conductivity, chemical and thermal stability, and hardness.^1−3^ They have also been increasingly studied for application in heterogeneous catalysis, as supports or as catalysts themselves,^4,5^ in electrocatalysis,^6−8^ and in energy storage.^9−11^ The use of carbides as catalyst supports has focused on supporting precious metals for applications in electrocatalysis, particularly water splitting, where materials must be stable at extremes of pH. There is a need for supported platinum group metals such as Pt and Ir catalysts to disperse the active phase and minimize precious metal use if large-scale implementation is to be realized.^12^ In water splitting, unsupported iridium-based catalysts struggle to make highly conductive continuous layers below 0.3–0.5 mg_Ir_ cm^–2^, after which the conductivity drops off rapidly due to a decline in layer quality.^13,14^ Supporting iridium oxide onto a substrate lowers the skeletal density of the catalyst, meaning that for equivalent iridium loading, the layer will be thicker, making it is easier to control the layer quality. In addition, if this substrate contributes to electronic conductivity, it can aid in achieving high utilization of the iridium in that layer. Therefore, supported catalysts can enable the lower loading anode layers needed to achieve the desired Ir-specific power density targets. It should be noted that Ir-based catalysts give the best balance between activity and stability: base-metal oxides are not stable in acid conditions,^15^ and while ruthenium oxide is more active, it also is not stable enough to give viable catalysts in practical applications.^16^

**Scheme 1:**
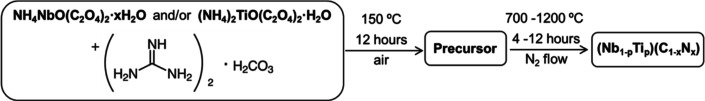
Synthesis Procedure for Nanostructured Niobium Titanium Carbonitrides

Tackett et al. deposited platinum on niobium carbide thin films to yield catalysts with high hydrogen evolution activity that were stable in both acidic and alkaline electrolytes.^17^ Stamatin and Skou supported platinum on niobium carbonitride for the oxygen reduction reaction (ORR) in acid electrolyte, finding that the interaction between the electrocatalyst and the support was better than for activated carbon supports, giving greater stability although with lower mass activity.^18^ Nam et al. studied partially oxidized niobium carbonitrides for nonplatinum catalysts in the ORR in acidic conditions, finding that an ORR onset was much higher than when pure niobium carbonitride was used.^19^ Li et al. loaded titanium nitride with iridium to catalyze the oxygen evolution reaction (OER), observing enhanced dispersion and inhibited aggregation of the nanoparticles, to give catalytic performance suitable for water electrolyzers.^20^ Avasarala et al. showed that nanoparticulate titanium nitride provides a catalyst support for proton exchange membrane (PEM) fuel cells that has better activities in acidic media than conventional platinized carbon electrocatalysts.^21^ Jin et al. studied titanium nitride and titanium carbonitride with hierarchical structures for the ORR in alkaline media and found activities comparable to Pt on carbon but with greater durability.^22^

The synthesis of carbides and carbonitrides typically uses high temperatures and reactive gases.^23^ This means that scalable preparation of materials to useful quantities for screening properties and ultimately manufacture presents challenges, which hinders their practical application. Furthermore, the use of high temperatures in synthesis generally yields large crystallites of materials with a low surface area, which are not useful for heterogeneous catalysts or as catalyst supports. Ideally, nanostructured materials are required. Traditionally synthesis uses a temperature-programmed carburization of transition metal oxide precursors using a gas mixture such as 20 (v/v) % CH_4_/H_2_.^24,25^ Niobium carbide cannot be produced below 950 °C by this approach,^24,25^ and these conditions result in samples that possess low specific areas.^26^ A different method for the synthesis of niobium carbonitride was reported by Chagas et al., who used lower temperatures and a helium environment.^27^ This route uses guanidine carbonate salt and ammonium niobate (V) oxalate hydrate as precursors that are heated at 150 °C in air for 12 h, followed by treatment at 400 °C for 4 h under helium and then at 450–900 °C for 2 h under helium. In this paper, we describe a simplified method based on this to provide a convenient synthesis approach to nanostructured niobium carbonitride and extend it to allow access to mixed titanium–niobium and pure titanium analogues. The materials are fully characterized using a range of experimental techniques, and the niobium carbonitrides are shown to have favorable properties as supports for iridium for oxygen evolution electrocatalysis in acid electrolytes, of importance in applications such as electrolyzers for splitting of water. A previous study of Ir-loaded NbC in this application by Karimi and Peppley showed excellent electrical conductivity, however, with poor performance for the OER, which was likely due to the poor surface area of their commercially sourced material.^28^ Our aim was to prepare materials with higher surface areas by a synthesis method, avoiding reactive gases and extreme temperatures. We have tested the optimum material as a support for iridium and examined its stability under acidic conditions, which is important for application in PEM devices, such as electrolyzers for water splitting. Acidic membranes (PEMs) are favored for high energy density devices because they allow higher power efficiency and longer membrane durability than alkaline membrane systems and can give higher purity gases. Acidic, proton-conducting membranes are also used in PEM fuel cells, but many conventional carbon supports are not suitably stable under acidic conditions, particularly when unexpected swings in potential are experienced.^29−31^

## Experimental Section

Ammonium niobate(V) oxalate hydrate (NH_4_NbO(C_2_O_4_)_2_·*x*H_2_O) was provided by CBMM with thermogravimetric analysis (TGA) used to calculate the degree of hydration (*x*) of each batch used. Guanidine carbonate salt (guanidinium carbonate, NH_2_C(=NH)NH_2_·1/2H_2_CO_3_) was provided by Sigma-Aldrich (99%) and ammonium titanyl oxalate monohydrate ((NH_4_)_2_TiO(C_2_O_4_)_2_·H_2_O) by Acros Organics (98%).

The synthesis of niobium carbonitrides was optimized using a two-step approach (Scheme 1):

1. A 1:5 molar ratio of ammonium niobate(V) oxalate hydrate (NH_4_NbO(C_2_O_4_)_2_·*x*H_2_O) (7.6 g) and guanidine carbonate (6.4 g) was ground for 5 min in a pestle and mortar. The mixture was heated in air for 12 h at 150 °C.
2. The product of step 1 was heated under a nitrogen flow to a selected temperature (700–1200 °C) using a ramp rate of 10 °C min^–1^ for 4–12 h, followed by cooling to room temperature.

This approach was further modified to use ammonium titanyl oxalate monohydrate in place of the ammonium niobate(V) oxalate hydrate to investigate the synthesis of titanium carbonitrides. The formation of mixed metal titanium and niobium carbonitrides was also studied by replacing some of the ammonium niobate(V) oxalate hydrate with ammonium titanyl oxalate monohydrate, with the molar ratio of precursors chosen to give a 1:1 ratio of Nb/Ti. For all materials, after cooling, the gastight seals of the tube furnace were slowly loosened over several hours before the nitrogen flow was terminated, and then the mixture was left for a further hour. This was done with the aim of passivating the surface of the materials to avoid the formation of pyrophoric powders and to allow handling the black powder product under ambient conditions.

Powder X-ray diffraction (PXRD) was measured at room temperature using either a Siemens D5000 diffractometer (Cu Kα_1/2_ radiation) in Bragg–Brentano geometry or a Panalytical X’Pert Pro MPD (Cu Kα_1_) radiation with a PIXcel solid-state detector. Pawley refinements were performed for the refinement of the unit cell of a material from powder XRD using TOPAS software implemented with jEdit.^32,33^ Combustion analysis for CHN was performed by Medac Ltd., UK. Surface area measurements were performed using a Micromeritics Tristar 3000 porosimeter. Samples were degassed before the measurements at 200 °C under nitrogen to eliminate any surface water or other volatile impurities. The nitrogen adsorption isotherm was recorded, and the surface area measurement was obtained from a seven-point linear fit using Brunauer–Emmet–Teller (BET) theory.

Transmission electron microscopy (TEM) was performed using a JEM 2800 (scanning) transmission electron microscope (STEM) operating at 200 kV. Bright-field imaging mode was carried out using a charge-coupled device (CCD). High-magnification lattice resolution imaging was carried out using CCD dark-field (Z-contrast) imaging in scanning mode using an off-axis annular detector. The scanning electron signal was acquired simultaneously with the other STEM images providing topological information on the sample. Compositional analysis was performed by X-ray emission detection in scanning mode.

Powder conductivity of powdered samples was measured at the Johnson Matthey Technology Centre, Sonning Common, UK using a custom-built apparatus. The material was placed in a cylinder between two gold-plated electrode plates (1 cm^2^) and compressed at 2 bar pressure under nitrogen using a piston. The thickness of the resulting pellets was measured, and the conductivity was determined from the measured resistivity from a change in voltage with applied current using an Autolab potentiostat. TGA was performed in air to 900 °C at 10 °C min^–1^ using a Mettler Toledo TGA/DSC 1–600 instrument.

XPS measurements were made using a Kratos Axis Ultra DLD spectrometer. Small amounts of powdered samples were mounted on electrically conductive carbon tape on a sample bar and loaded into the spectrometer with a base pressure below 1 × 10^–10^ mbar. A monochromated Al Kα X-ray source was used to illuminate the sample, and measurements were made at room temperature with a takeoff angle of 90° with respect to the surface parallel. Core-level spectra were recorded from an analysis area of 300 × 700 μm^2^ with a pass energy of 20 eV (resolution ca. 0.4 eV). The spectrometer work function and binding energy scale were calibrated using the Fermi edge and 3d_5/2_ peak of a polycrystalline Ag sample. The surface was flooded with a beam of low-energy electrons throughout the experiment to prevent surface charging. This necessitated recalibration of the binding energy scale which was done using the C–C/C–H component of the C 1s spectrum, referenced to 284.8 eV. The spectra were analyzed using the CasaXPS package,^34^ with Shirley backgrounds and mixed Gaussian–Lorentzian (Voigt) lineshapes. The analyzer transmission function was determined using clean metallic foils to determine the detection efficiency across the full binding energy range for compositional analysis.

Inductively coupled plasma optical emission spectroscopy (ICP-OES) was performed using a Thermo 7600 radial ICP-OES spectrometer. Between 20 and 30 mg of sample in duplicate was digested in Zr crucibles using sodium peroxide fusion acidified with 15 mL of HCl and 1 mL of HF. Samples were made up to 100 mL with deionized water before analysis.

The Polaris diffractometer at the ISIS Neutron and Muon Source, UK, was used for neutron diffraction measurements.^35^ Vanadium cans of 6 or 8 mm diameter were used to contain the samples, which were placed inside the evacuated instrument chamber. Rietveld refinements of crystal structures were performed against the neutron diffraction data with the GSAS software,^36^ visualized using the EXPGUI interface.^37^

Iridium loading on the carbonitride materials was performed by a method based on that of Karimi and Peppley.^28^ 0.5 g of carbonitride was stirred in 100 mL of ethylene glycol with 0.5 g NaOH. Chloroiridic acid (H_2_IrCl_6_) was added to give a target of 30 wt % iridium. The slurry was heated to 160 °C and stirred for 2 h, and after cooling, 1 M H_2_SO_4_ was added to reach a pH of 2. The solid was recovered by filtration and washed 3 times with 100 mL of deionized water, before being dried at 80 °C. The same method was used to load iridium on carbon of a similar surface area (graphitized Ketjen black) to provide a reference material. To provide a further reference material a sample of iridium oxide, IrO_*x*_, was used, as provided by Alfa-Aesar (Premion). To fabricate electrodes, 0.1 g of catalyst (Ir on Nb(C,N), Ir on carbon, or IrOx) was combined with 0.02 g of aqueous Nafion solution (11.92 wt % solids) and 3 drops of water and the mixture homogenized in a planetary mixer for 15 s at 3000 rpm. This was then shear-mixed in a planetary mixer for a further 2 min at 3000 rpm using 5 mm diameter yttrium stabilized zirconia ceramic beads with further water added to aid mixing, with manual stirring to disperse any sediment. The resulting ink was further diluted by adding 2 g of water and then sprayed onto a 7 × 7 cm^2^ square of Toray paper (hydrophobic gas diffusion layer TGP-H-060, a carbon fiber composite paper) using 0.25 mL of the ink, diluted with 0.75 mL of isopropanol and 1.5 mL of water in a spray gun with the Toray paper placed on a hot plate at 80 °C. The resulting layer was weighed to obtain an approximation of its thickness, aiming for 0.05–0.15 mg cm^–2^. Accurate loadings and the distribution of iridium were measured with a desktop X-ray fluorescence (XRF) instrument FISCHERSCOPE X-RAY XDV-SDD. 20 mm diameter buttons were cut from the resulting layer, and these showed typically 0.2 mg cm^–2^ of iridium.

The activity of the catalysts was assessed by wet cell testing in 0.1 M H_2_SO_4_ at 60 °C degassed with nitrogen. A button loaded with the sample was immersed in 200 mL of 0.1 M H_2_SO_4_ overnight under vacuum, and 5 mL of the soaking solution was retained for ICP–MS analysis. A gold wire was attached to the button as the working electrode. The wet cell was filled with 100 mL of 0.1 M H_2_SO_4_, and once the button was in place, a 5 mL sample was taken for ICP–MS analysis, before replacing the solution with fresh 0.1 M H_2_SO_4_. The counter electrode was a Pt wire, and the reference electrode was the reversible hydrogen electrode (RHE), with hydrogen bubbled over a Pt/C catalyst. The cell was first cycled between 0 and 1.35 V vs RHE at various scan rates (300–5 mV s^–1^), followed by an activity sweep between 1 and 1.55 V vs RHE at 1 mV s^–1^ at the beginning of life (BOL). A sample of 5 mL was then taken for ICP–MS analysis and replaced with fresh 0.1 M H_2_SO_4_. A degradation cycle was performed between 0.6 and 1.35 V vs RHE at 100 mV s^–1^ for 1000 cycles (∼4 h 10 min). This was selected to bring the potential up to the onset of OER, while not forming bubbles of oxygen which could affect the reliability of the results. Another 5 mL sample was taken for ICP–MS analysis. An end-of-life (EOL) activity test was then performed after the degradation cycles equivalent to the initial scan. The mass activity (A/g) was calculated as the mass activity of iridium.

## Results and Discussion

**Figure 1:**
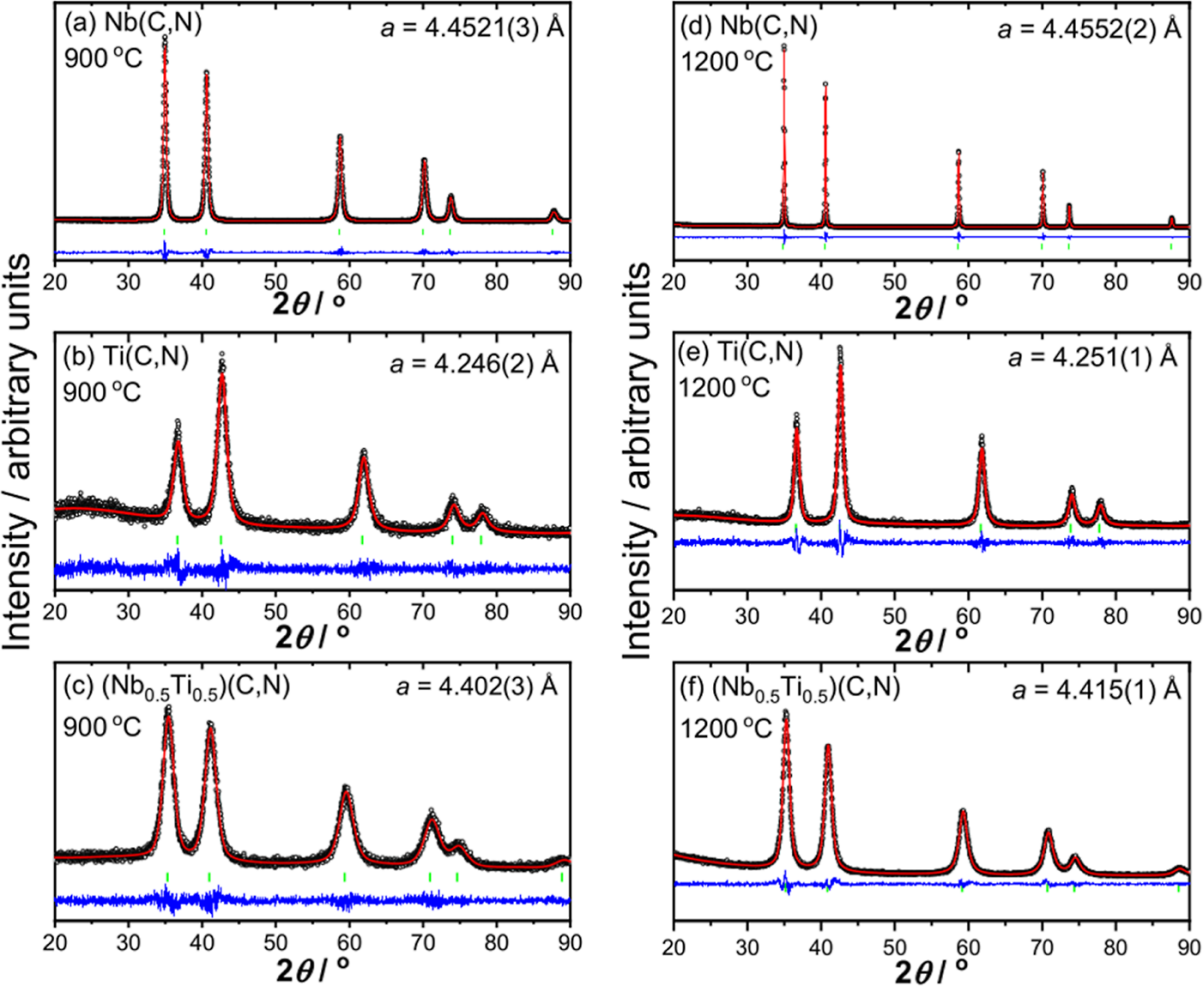
Profile fitted powder XRD patterns of samples of Nb(C,N), Ti(C,N), and (Nb_0.5_Ti_0.5_)(C,N) prepared at (a–c) 900 °C and (d–f) 1200 °C. The circles are the measured data (either Cu Kα_1_ or Cu Kα_1/2_ radiation), the red line is the result of profile fitting, the blue line is the different curve, and the green ticks are the allowed positions of the cubic *Fm*3̅*m* unit cell. The refined cubic lattice parameter is indicated for each pattern.

The synthesis route used in this work is based on that reported by Chagas et al., who studied the formation of niobium carbonitrides and used them as catalysts for hydrodesulfurization of dibenzothiophene.^27^ Herein, we show that the synthesis method can be simplified and then extended to include mixed-metal carbonitrides, specifically those of titanium and niobium. In the original work, a three-step heat treatment of mixtures of ammonium niobium oxalate and guanidine carbonate in a helium atmosphere was used.^27^ We found that the process could be simplified to a two-step process with nitrogen replacing helium, where an initial heat treatment in air at 150 °C for 12 h followed by heating under a flow of nitrogen to various temperatures (700–1200 °C) for periods between 1 and 24 h was sufficient to produce crystalline materials. Initially, the preparation of the three materials Nb(C,N), Ti(C,N), and (Nb_0.5_Ti_0.5_)(C,N) was tested at 900 °C and at 1200 °C with 4 h reaction time. Analysis of the powders formed by powder XRD showed characteristic peaks of cubic *Fm*3̅*m* rock-salt materials, with no evidence for any crystalline impurities, but with a broadened diffraction profile indicative of nanostructure (Figure 1). For Nb(C,N) (Figure 1a,d), the refined cubic lattice parameters are between the values expected for crystalline NbC (4.469 Å)^38^ and NbN (4.394 Å).^39^ The titanium carbonitride also shows only Bragg peaks for the expected cubic *Fm*3̅*m* unit cell, Figure 1b,e. These materials were found to be more difficult to prepare without significant oxidation of the surface, often occurring spontaneously after the samples were removed from the tube furnace, evidenced by the appearance of a white coating on the otherwise black powder. The titanium materials also appear to have an amorphous material present, particularly evident between 20 and 35° in the powder XRD pattern where there is an observable extra background (Figure 1b,e). The fitted lattice parameters of the titanium carbonitrides are closer to TiN (*a* = 4.235 Å)^40^ than TiC (*a* = 4.328 Å).^41^

Using mixtures of ammonium niobate oxalate hydrate and ammonium titanyl oxalate monohydrate in combination with guanidine carbonate, it proved possible to prepare mixed niobium–titanium carbide materials. The powder XRD patterns of (Nb_0.5_Ti_0.5_)(C,N) materials prepared at two temperatures are shown in Figure 1c,f. Here, a 1:1 ratio of the two metal precursors was used in synthesis, and the refined lattice parameters lie between the values seen for TiN and NbC, although closer to the latter. The only other report of the quaternary system Ti–Nb–C–N used zone melting and annealing of metals and binary carbides under nitrogen and produced materials with bulk cubic structures but deficient in carbon and nitrogen.^42^

For each of the 3 compositions, with increasing temperature of syntheses, the Bragg peaks become sharper, indicating an increase in crystallinity, and Scherrer analysis gives average crystal domain sizes from ∼30 nm for the samples prepared at 900 °C to ∼60 nm for the samples prepared at 1200 °C.

**Figure 2:**
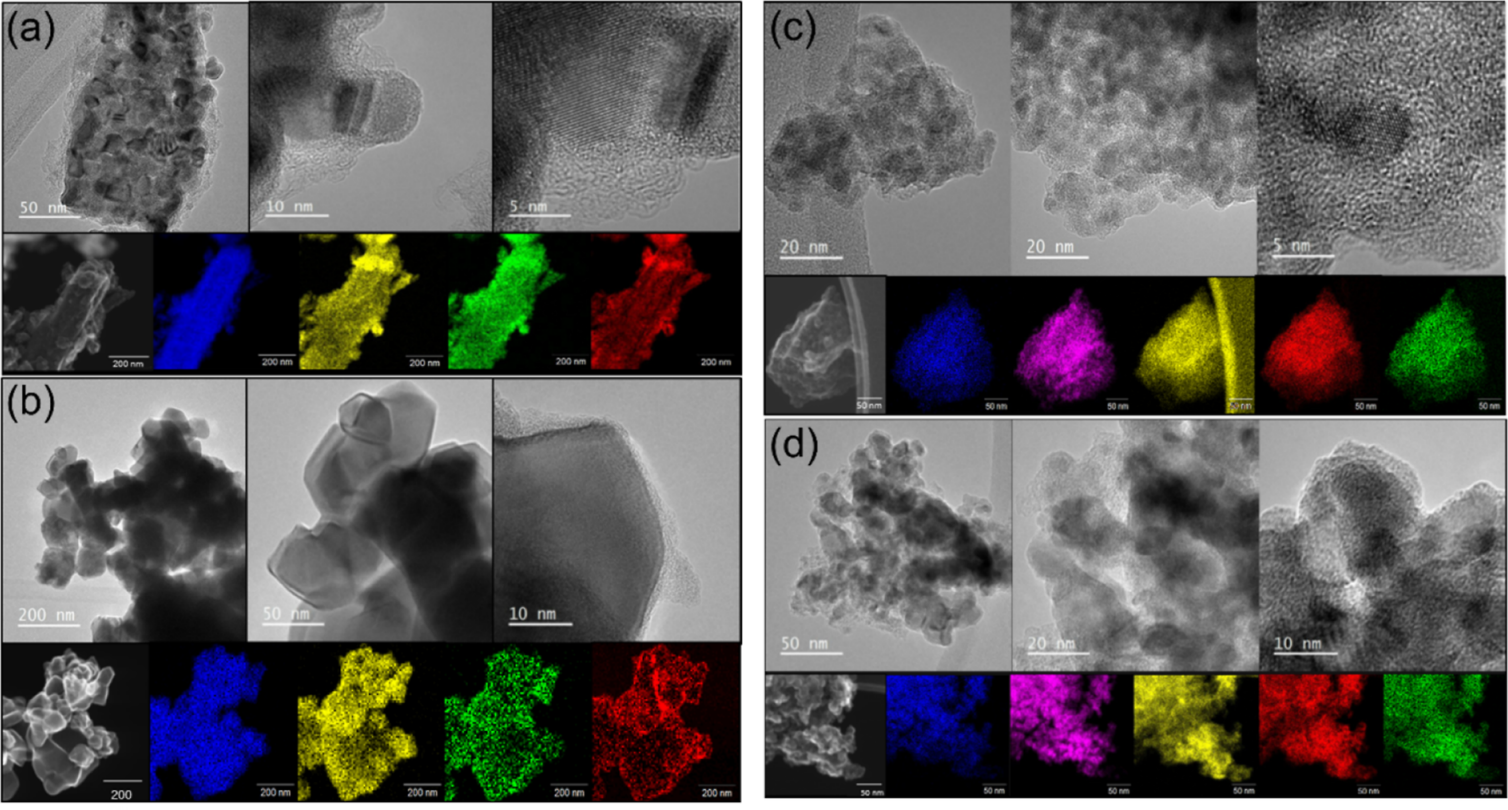
TEM and EDS element maps for (a) Nb(C,N) prepared at 900 °C, (b) Nb(C,N) prepared at 1200 °C, (c) Nb_0.5_Ti_0.5_(C,N) prepared at 900 °C, and (d) Nb_0.5_Ti_0.5_(C,N) prepared at 1200 °C. Nb in blue, Ti in pink, C in yellow, N in green, and O in red. Note that the TEM grid is carbon.

Given the difficulty in preparing samples of the pure titanium materials due to the rapid surface oxidation, a more detailed analysis was performed on the niobium and mixed niobium–titanium materials. TEM images were recorded to assess sample morphology on the nanoscale and to allow local atomic-scale elemental analysis. Figure 2a,b shows the results obtained for typical Nb(C,N) materials prepared at two temperatures. At the lower temperature of synthesis, the material consists of individual crystallites of less than 10 nm diameter that are highly agglomerated, while at the higher synthesis temperature, the crystallites become better defined with evidence for crystal growth giving large particles of ∼50 nm in dimension. The EDS mapping shows little evidence for excess surface carbon, even for the smallest particles where only a little amorphous material is apparent surrounding the individual crystallites. Instead, nitrogen and oxygen are detected equally along with niobium. The presence of oxygen would suggest some surface oxidation, although no oxide phases were detected by powder XRD. In the case of the mixed-metal materials, TEM shows the same increase in crystallite size with an increased temperature of synthesis, Figure 2c,d, while the EDS maps show an even distribution of the two metals, with no evidence of phase separation.

In order to assess the possibility of using the materials in the desired application as acid-resilient catalyst supports, we examined their stability toward exposure to strongly acidic solutions. After immersion in 1 M H_2_SO_4_ at 80 °C for 24 h, powder XRD was initially used to examine any changes in crystallinity (Figure S1, Supporting Information). Note that these conditions are more extreme than those used in electrochemical tests but were designed to assess the robustness of the materials and to select the best for application in PEM devices. This shows how the niobium carbonitride material retains its crystallinity: the cubic lattice parameter changes little with only some increase in peak broadening. On the other hand, the mixed titanium–niobium material is evidently degraded with a significant loss of crystallinity and the appearance of a broad low-angle feature between 20 and 30° 2θ, indicating some amorphization of the sample. ICP-OES analysis of the solutions after this acid treatment reveals that little leaching of Nb has occurred (less than 1.5% of the total), whereas for the niobium–titanium materials, ∼15% of the Ti is lost into the solution, which suggests that it is the instability of Ti that drives the collapse of the mixed-metal material.

Given the acid instability of materials prepared using titanium, the pure niobium materials were the focus of in-depth characterization and assessment of their properties. TGA was used to estimate the amount of amorphous carbon (or carbon nitride) present in the materials. The combustion of metal carbide-nitrides is expected to result in an increase in mass since the oxide product(s) have a larger formula weight than the starting material, e.g., the combustion of niobium carbonitride, with carbon contaminant, can be written as
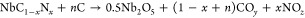


**Figure 3:**
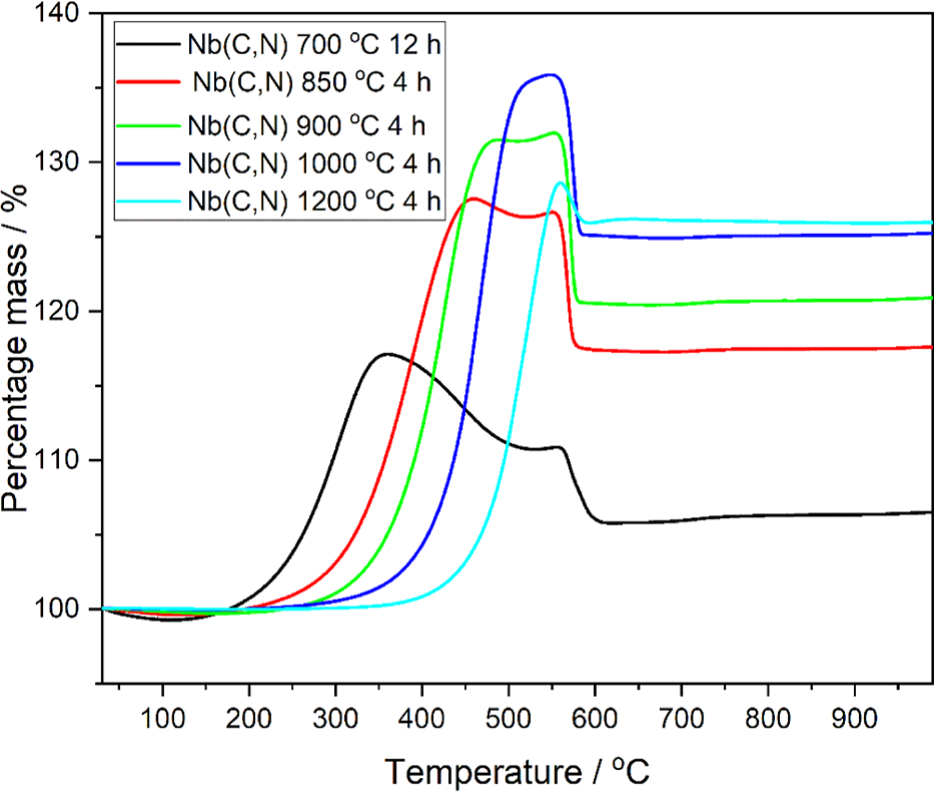
Thermogravimetric analysis of niobium carbonitride materials prepared under different conditions.

Figure 3 shows the TGA results for Nb(C,N) materials prepared at various temperatures and times. The TGA for all materials shows a characteristic large mass increase on heating in air to moderate temperatures (200–500 °C) followed by a mass loss before the final resting mass is observed above 600 °C. The initial increase in mass is likely due to oxidation and might imply the formation of carbonate before calcination to form an oxide product and has been observed before for NbC materials prepared by other methods.^28^ It is also worth noting that for the materials prepared at lower temperatures, the onset of decomposition occurs at lower temperature. This suggests that the higher surface area materials are oxidized more readily. In order to quantify the TGA results, the bulk C/N content, as determined separately from combustion analysis, was used along with powder XRD identification of the combustion product at 800 °C (Figure S2, Supporting Information). This allowed the determination of the mass percentage of amorphous carbon present in the materials (assuming it is pure carbon), and the results are summarized in Table 1. It is noteworthy that the amount of amorphous carbon is extremely low, even for the sample prepared under the mildest conditions, and once a temperature of 1000 °C is used, the amount detected is negligible. Further TGA coupled with different scanning calorimetry and mass spectrometry reveals that the initial increase of mass during heating is accompanied by the removal of O_2_ from the air flow, while no significant release of CO_2_, NO_2_, or NO takes place until considerably higher temperatures, and that both events are accompanied by sharp responses in the DSC (Figure S3).

**Table 1:** Analysis of Nb(C,N) Materials: The CHN Data to Give Ratios of Carbon/Nitrogen, and the Amount of Amorphous Carbon Present in Each Sample, Along with BET Surface Areas and Powder Conductivity

synthesis temperature/°C	elemental analysis			amorphous carbon/wt %	BET surface area/m^2^g^–1^	powder conductivity/S cm^–1^
	Nb	C	N			
700	1	0.47	0.53	2.18	a	0.010
850	1	0.61	0.39	0.97	72.6	1.29
900	1	0.63	0.37	0.76	51.8	4.82
1000	1	0.67	0.3	0.00	14.5	24.1
1200	1	0.61	0.32	0.00	4.44	31.6

a The BET surface area was not determined because of the comparatively higher amorphous carbon content of this sample.

BET surface areas measured from nitrogen adsorption isotherms are also included in Table 1. These show how increasing the synthesis temperature of the pure niobium materials results in a lowering of surface area, while the mixed niobium–titanium material has a notably higher surface area. The powder conductivity is only an approximation of the bulk conductivity of the materials since it neglects grain boundary effects, but the measured values show appreciable conductivity, similar to carbon materials measured using the same apparatus. This indicates their suitability as conducting catalyst supports.

**Figure 4:**
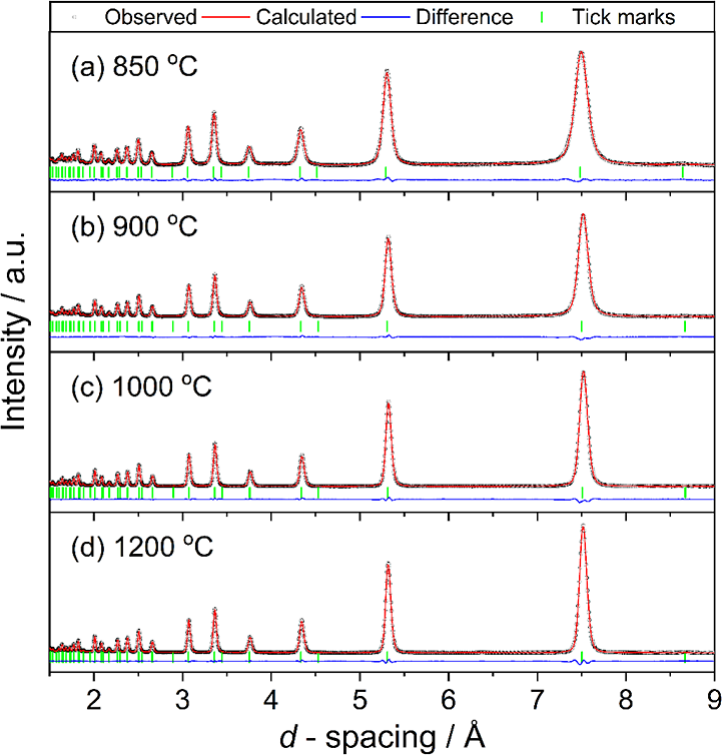
Final Rietveld fits (space group *Fm*3̅*m*) of neutron diffraction patterns from Bank 3 of the POLARIS diffractometer of Nb(C,N) made at different temperatures for 4 h. See Table 2 for refined parameters.

Powder neutron diffraction was used to determine crystal structures, including chemical composition, since the neutron scattering lengths of C and N (*b*_coh_ = 6.646 fm and 9.360 fm, respectively^43^) differ sufficiently to refine their proportion. The refinement of site occupancies was approached initially by fixing the total C + N content to be equal to 1 but allowing the proportion of each to vary with isotropic temperature factors (*U*_iso_) fixed at reasonable values. Then, the constraints were released, and all parameters, except Nb site occupancy, were allowed to vary in a final cycle. Figure 4 shows the final Rietveld fits to the neutron diffraction patterns of samples prepared at five different temperatures, and Table 2 contains the refined chemical compositions.

**Table 2:** Rietveld Fitted Parameters from Neutron Diffraction Patterns of Nb(C,N) Prepared at Different Temperatures for 4 h

synthesis temperature (°C)	lattice parameter/Å	Nb		C	N	C and N	refined composition
		Occ.	*U*_iso_ × 100/Å^2^	Occ.	Occ.	*U*_iso_ × 100/Å^2^	
850	4.44587(2)	1	0.323(1)	0.571(1)	0.429(1)	0.666(2)	NbC_0.57_N_0.43_
900	4.45116(1)	1	0.400(1)	0.599(1)	0.397(1)	0.372(1)	NbC_0.60_N_0.40_
1000	4.45494(1)	1	0.449(1)	0.655(1)	0.332(1)	0.272(1)	NbC_0.66_N_0.33_
1200	4.45333(1)	1	0.476(2)	0.620(1)	0.359(1)	0.229(1)	NbC_0.62_N_0.36_

The Rietveld fitting shows an increase in unit cell size with increasing temperature of synthesis to 1000 °C, which is consistent with an increase in carbon content when considering the literature values of lattice constants for NbC and NbN (see above). The refined ratio of carbon/nitrogen confirms this, with an increase in reaction temperature. The unit cell size slightly decreases for materials in the 1200 °C synthesis, and the refinement of the carbon/nitrogen ratio suggests that this is due to a deficiency at the carbon/nitrogen site. It can be noted that there is no evidence of carbon/nitrogen ordering from the neutron crystallography. Comparing the refined values of carbon/nitrogen content from neutron diffraction with those of bulk chemical analysis (Table 1) shows broad agreement, but it should be kept in mind that the fitting of powder diffraction considers only the crystalline portion of the sample and not any amorphous components, nor any surface composition of small particles.

**Figure 5:**
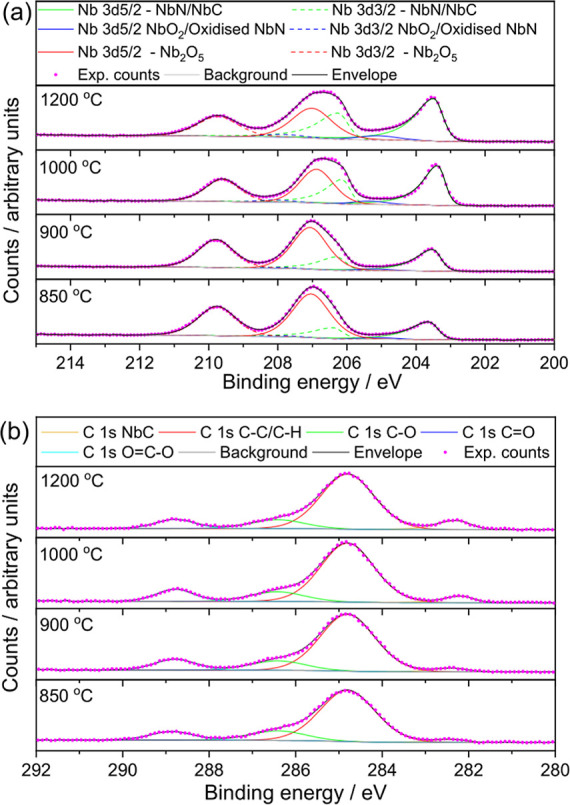
XPS analysis of Nb(C,N) materials in the (a) Nb 3d region for samples prepared using various synthesis temperatures at 4 h and in the (b) C 1s region for samples prepared using various synthesis temperatures at 4 h. The top panels indicate the assignment of the various features (see also Table 3).

X-ray photoelectron spectroscopy was used to analyze surface chemistry and composition. For the niobium carbonitride materials although the surface contains a high proportion of carbon, a significant amount of oxygen is also present, more so than nitrogen, which is a minor component (Table 3). The proportions of each did not change significantly between samples. This would be consistent with some surface oxidation and accumulation of adventitious carbon species on the surface. Indeed, analysis of the Nb 3d region of the XPS spectra (Figure 5a) shows a majority signal characteristic of a Nb_2_O_5_-like species in all samples, along with an increasing proportion of signal that can be assigned as NbC or NbN as synthesis temperature is increased. A minor component can be assigned as NbO_2_ or oxidized NbN. The full assignment of the spectral features is given in Table S1. Since no crystalline niobium oxides were detected by powder XRD, the oxidized niobium species observed by XPS can only be present as the nanostructure at the surface.

**Table 3:** Surface Composition of Nb(C,N) Materials from the Analysis of XPS

synthesis temperature/°C	Nb %	C %	O %	N %	Na %
850	11.5	51.5	32.7	2.8	1.6
900	10.0	57.3	29.9	2.1	0.6
1000	9.8	61.1	27.1	2.1	not detected
1200	14.4	52.6	29.6	3.5	not detected

The carbon 1s region of the XPS is very similar for all samples and shows a variety of environments, including some majority of C–C bonds and some oxidized carbon, with only a minority of NbC-like carbon (Figure 5b and Table S1). This confirms the presence of a carbon-rich surface, despite the lack of significant bulk carbon seen by TGA.

O 1s and N 1s regions of the XPS are shown in the Supporting Information, Figures S4 and S5. The N 1s region shows the presence of NbN, oxidized NbN, and organic nitrogen.^44,45^ There are larger amounts of NbN when a lower temperature of synthesis was used, while there are increasing amounts of oxidized NbN with increasing synthesis temperature. The O 1s region shows C=O, C–O, metal oxides, and either H_2_O or organic O in all the samples. The presence of C=O and C–O suggests that carbonate may be present at the surface.

**Figure 6:**
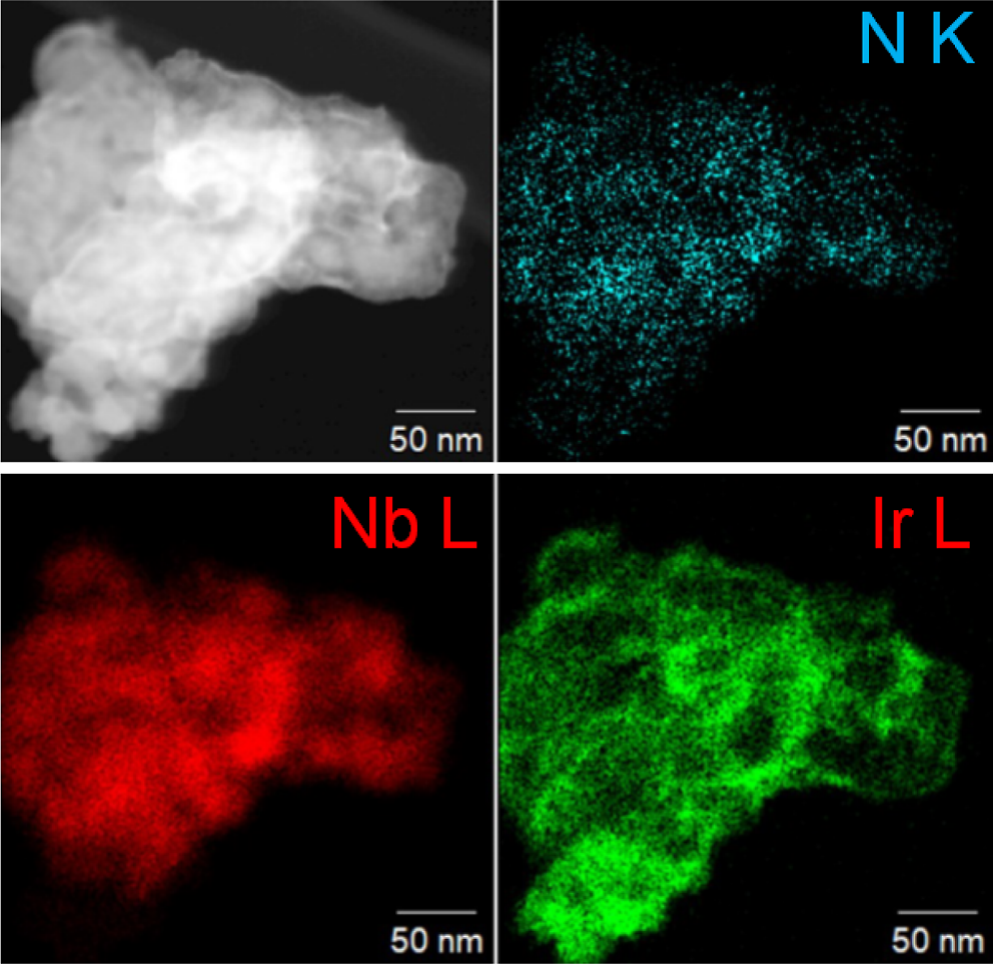
TEM image (top left) and EDS maps of iridium dispersed on Nb(C,N).

Having prepared acid-stable niobium carbonitride materials with surface areas suitable for use as catalyst supports, we explored their use as supports for iridium and assessed their properties toward electrocatalysis of oxygen evolution. The material prepared at 900 °C was selected since this has a reasonable surface area and only small amounts of amorphous carbon present. For comparison, a carbon of similar surface area was similarly loaded. The electrochemical behavior of the two iridium-supported electrodes was compared with an IrO_2_ material of similar surface area. Figure 6 shows a TEM image of the sample of Nb(C,N) loaded with iridium, illustrating the good dispersal of the precious metal on the nanoscale.

**Figure 7:**
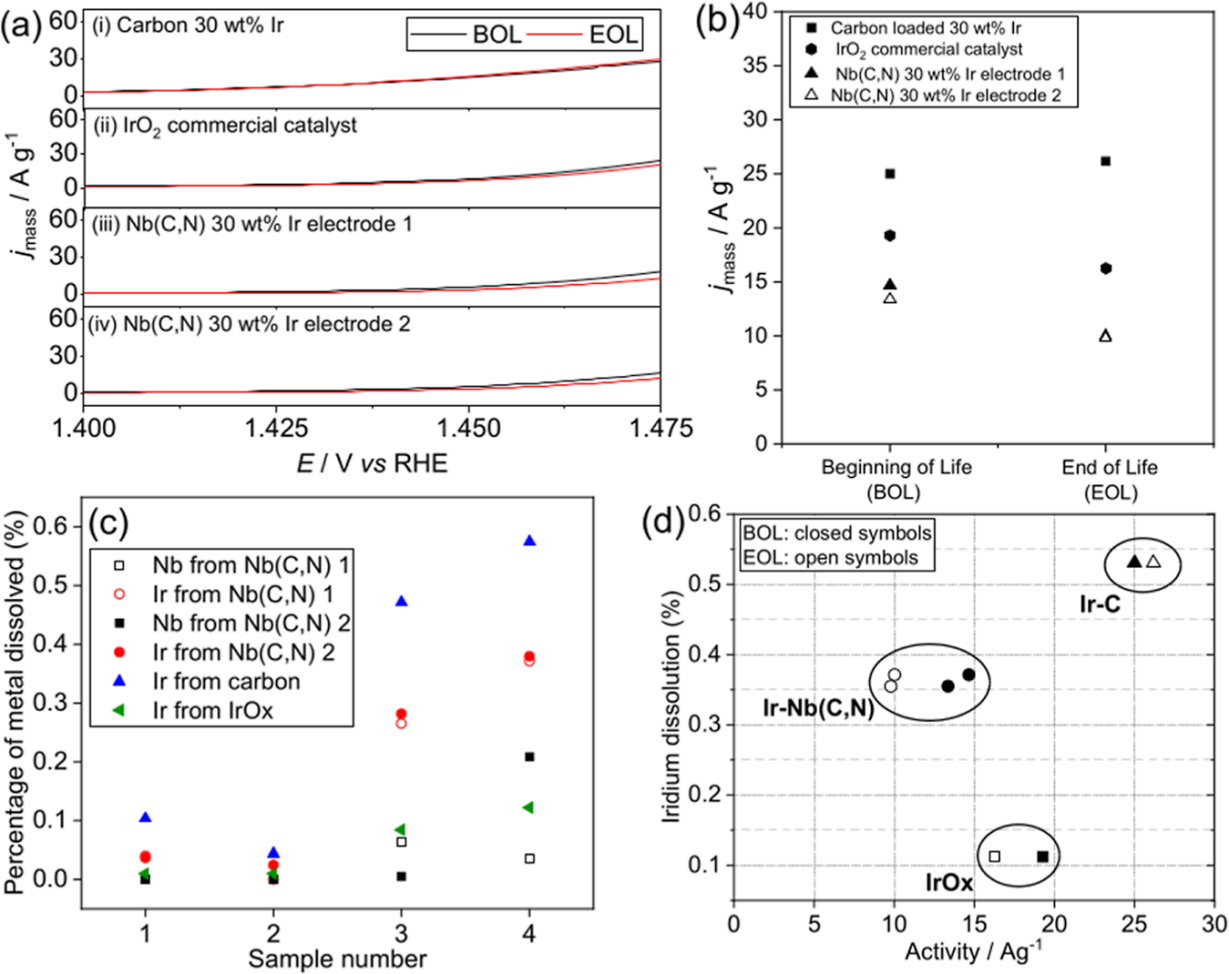
(a) Mass activity measurements comparing two Nb(C,N) electrodes loaded with 30 wt % iridium with carbon loaded with 30 wt % iridium and IrO_*x*_ commercial catalyst. (b) Mass activity measured at 1.47 V for the same materials. BOL = beginning of life, EOL = end of life. (c) Dissolution of Nb and Ir into solution, as measured by ICP for the two Nb(C,N) electrodes loaded with 30 wt % iridium, with sampling as discussed in the text, along with Ir dissolution for the Ir on carbon and the reference IrO_*x*_ material, (d) plot of activity beginning of life (BOL) and end of life (EOL) against the percentage of metal dissolved between samples 4 and 2.

Two electrodes of the carbonitride material were tested for reproducibility. The mass activity OER measurements are shown in Figure 7a, and the activity values at 1.47 V are shown in Figure 7b. The carbon loaded with 30 wt % iridium shows little change in activity after the last electrochemical cycling (end of life) and in fact a small increase. This is not unexpected as Ir is known to initially increase in activity as it activates to its more catalytically active surface oxides during the application of potential.^46^ The NbC_1–*x*_N_*x*_ loaded with 30 wt % iridium shows lower activity levels than both the commercial IrO_2_ and the carbon loaded with 30 wt % iridium; however, it still shows reasonable stability with an activity of around 10 A g^–1^ of iridium end of life. The moderate drop in activity of IrO_*x*_ and the Ir on Nb(C,N) may be due to degradation or restructuring of the catalysts.

It is well established that changes in the polarization curve alone are not a measure of a catalysts stability but that mass loss must also be considered.^47^ Elemental analysis of solution samples taken during the electrochemical testing was carried out using ICP–MS to examine the leaching of metals. Sample 1 was taken from the solution wetting the button overnight, sample 2 from the initial solution in the cell, sample 3 from after the first activity tests, and sample 4 from after the 1000 cycles in the cell at the end of life (Figure 7c). Less than 0.2% of the niobium dissolves from either of the electrodes tested, and less than 0.4% of the iridium dissolves. For comparison, for the carbon loaded with iridium, more than 0.5% dissolves into solution on the fourth sampling.

In Figure 7d, the mass activity with the percentage of iridium dissolved is plotted together. Here, the percentage of iridium dissolved is taken as the difference in percentage dissolved between samples 4 and 2 in the testing protocol, i.e., the difference between pre-electrochemistry and after 1000 cycles of testing. This clearly shows how Ir on carbon, while more active, actually suffers from the most severe loss of iridium into the solution. The Ir on niobium carbonitride offers compromise with less iridium loss but still an appreciable activity. The balance of activity and stability is important for use in practical applications. As expected, the iridium oxide reference material offers superior properties, in terms of the least dissolution of iridium; however, for implementation in a real device, supported catalysts are more desirable, for ease of layer preparation, thrifting of the precious metal, and control of dispersion of the active catalyst to optimize its properties,^12^ which points toward a benefit for of the niobium carbonitride support.

Comparison with related materials already reported in the literature shows the benefit of using Nb(C,N) with good surface area as a support for iridium. Karimi and Peppley used commercially supplied NbC with a stated particle size of 5 μm and measured BET surface area of 0.9 ± 0.3 m^2^ g^–1^ and loaded iridium (20 wt %) in a similar way to that used here, testing the catalyst for OER activity in 0.5 M H_2_SO_4_.^28^ It is difficult to compare mass activities directly with the previous study, since the values quoted there were per total mass of catalyst, rather than per mass of measured iridium content, but Karimi and Peppley compared behavior with TaC and TiC and noted that the surface area of the support was more important than its conductivity in giving good performance. The previous study also did not examine the dissolution of the support and iridium, as we have done here, and it should be noted that we are studying Nb(C,N) materials and not NbC, and differences in the surface chemistry are likely to play an important role in the interaction with iridium. Finally, it is worth pointing out that our materials are distinct from the two-dimensional MXene carbides that have been studied as catalysts and catalysts support, and the MXene Nb_2_C provides a comparison, for which its instability under oxidative and aqueous conditions,^48^ along with its complex synthesis that typically involves highly corrosive chemicals,^49^ makes it unsuited for the applications we have explored here for the rock-salt niobium carbonitride.

## Conclusions

We have optimized a convenient synthesis route to nanostructured carbonitrides of niobium, extended it to titanium carbonitrides and mixed-metal materials, by thermal decomposition under moderate temperature under a nitrogen atmosphere and characterized the bulk and surface chemistry of the most stable Nb(C,N) materials. As well as potentially being easily scalable, the route we have developed allows access to materials with good surface areas, making it suitable for the support of catalysts, as demonstrated in the case of iridium. The acid resilience of the samples towards dissolution is noteworthy, even under applied potential. Implementation in PEM devices, such as fuel cells and electrolyzers, to examine their stability under operating conditions and their interaction with other device components should be the topic of future work, and this should also include analysis of spent catalysts along with mechanistic studies. The method of iridium deposition should also be explored to optimize the support–catalyst interaction and to minimize the use of iridium, and the nature of the active iridium species should be investigated. Finally, while we find that the titanium carbonitrides are less stable with respect to oxidation, the synthesis method used here could conceivably be developed for the production of other nanoscale transition-metal carbides by the use of suitable oxalate precursors.

## Supporting Information Available

Further PXRD data, further thermogravimetric analysis, O 1s and N 1s regions of XPS, and full assignment of XPS (PDF)
