# Supplementary material for: Nanostructured Niobium and Titanium Carbonitrides as Electrocatalyst Supports
Source: ACS Appl Nano Mater. 2024 Apr 24;7(9):10120–9. doi: 10.1021/acsanm.4c00503 (PMC11091850; doi:10.1021/acsanm.4c00503)
Supplement: Supplementary file 1 — an4c00503_si_001.pdf [file an4c00503_si_001.pdf]

Supporting Information

Nanostructured Niobium and Titanium Carbonitrides as Electrocatalyst Supports

Lucy K. McLeod,[1,2] Geoffrey H. Spikes,[2] Christopher M. Zalitis,[2] Katie Rigg,[2] Marc Walker,[3] Helen Y. Playford,[4] Jonathan D.B Sharman[2] and Richard I. Walton[1*]

1. Department of Chemistry, University of Warwick, Gibbet Hill Road, Coventry CV4 7AL, UK
2. Johnson Matthey Technology Centre, Blounts Court, Sonning Common, Reading, RG4 9NH, UK
3. Department of Physics, University of Warwick, Gibbet Hill Road, Coventry CV4 7AL, UK
4. ISIS Neutron and Muon Source, Rutherford Appleton Laboratory, Didcot OX11 0QX, UK

*Author for correspondence: r.i.walton@warwick.ac.uk

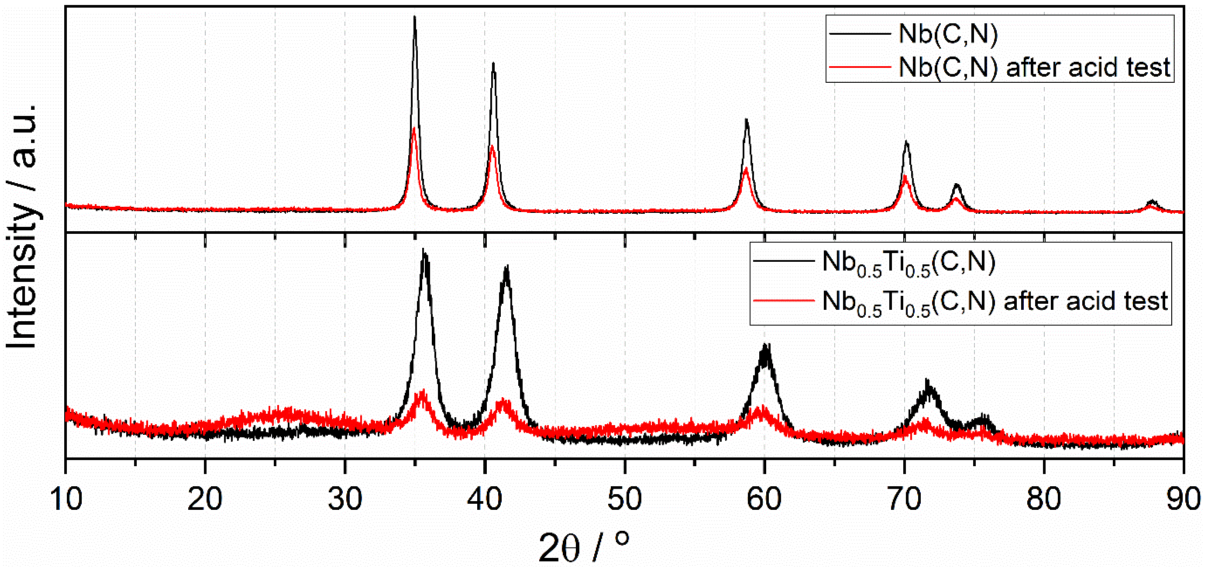

Figure S1: Powder XRD (Cu K$\alpha_{1/2}$ radiation) of samples of Nb(C,N) and $Nb_{0.5}Ti_{0.5}$(C,N) prepared at 900 °C before and after immersion in 1 M H_2SO_4 at 80 °C for 24 hours

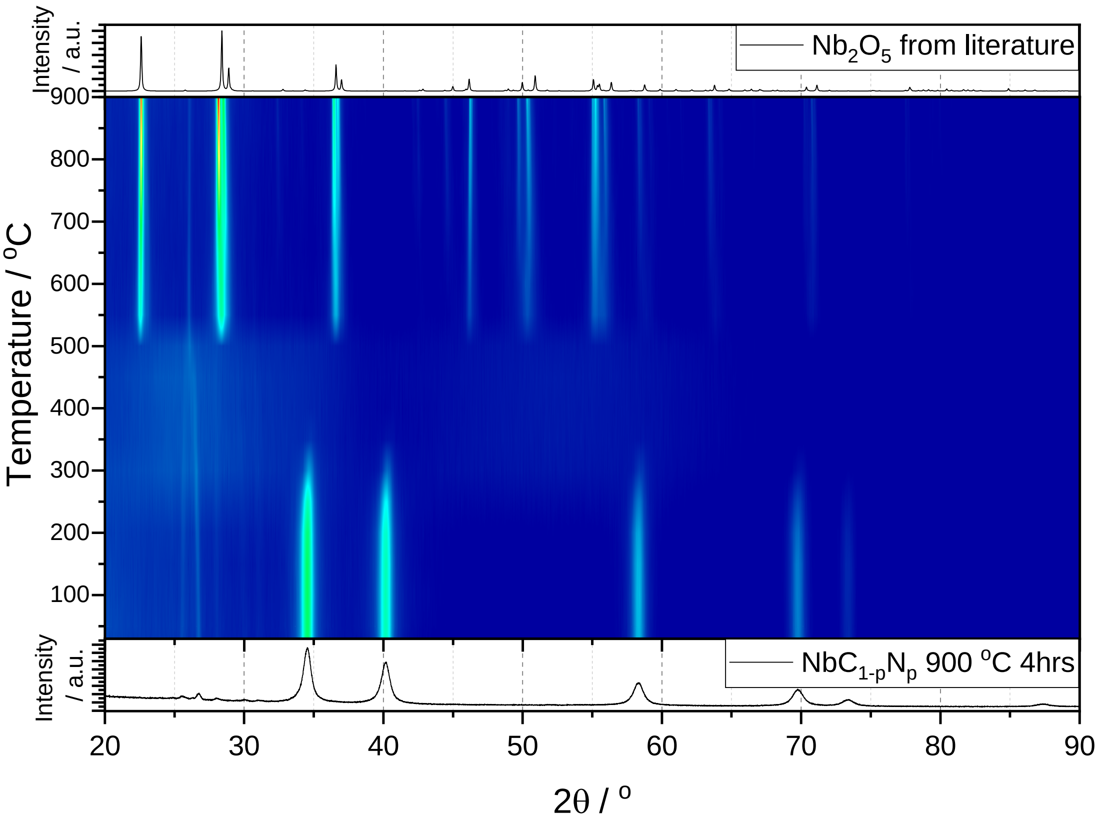

Figure S2: Powder XRD measured from Nb(C,N) prepared at 900 °C during heating in air to 800 °C (Bruker D8 diffractometer with Cu Ka radiation and Anton-Parr HTK900 heating stage). This shows the formation of the Nb_2O_5 product.

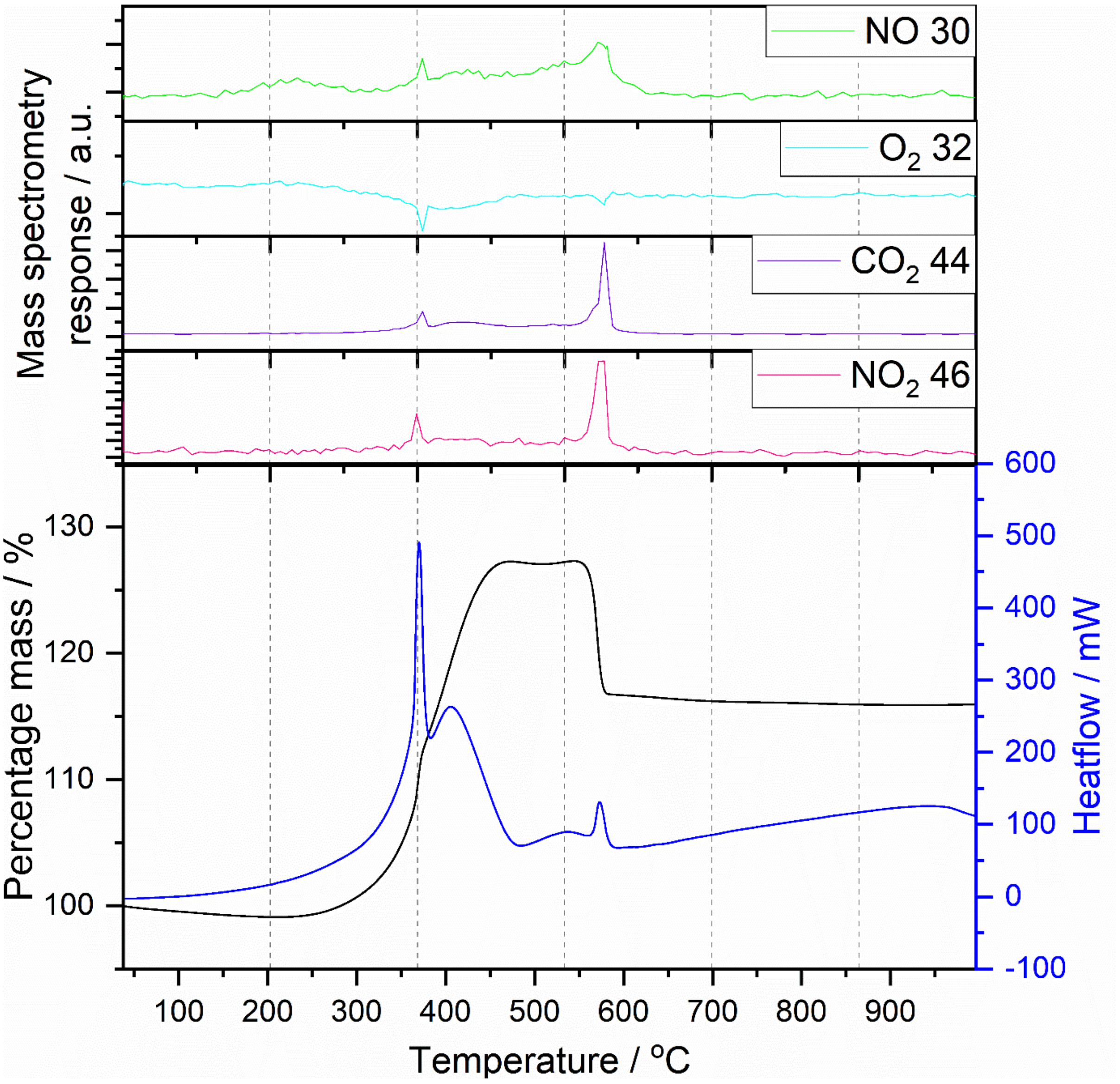

Figure S3: TGA-DSC-MS from Nb(C,N) prepared at 900 °C during heating in air to 1000 °C (). This shows the initial increase in mass is accompanied by uptake of oxygen from the air, while the later decrease in mass corresponds to combustion with release of carbon and nitrogen oxides.

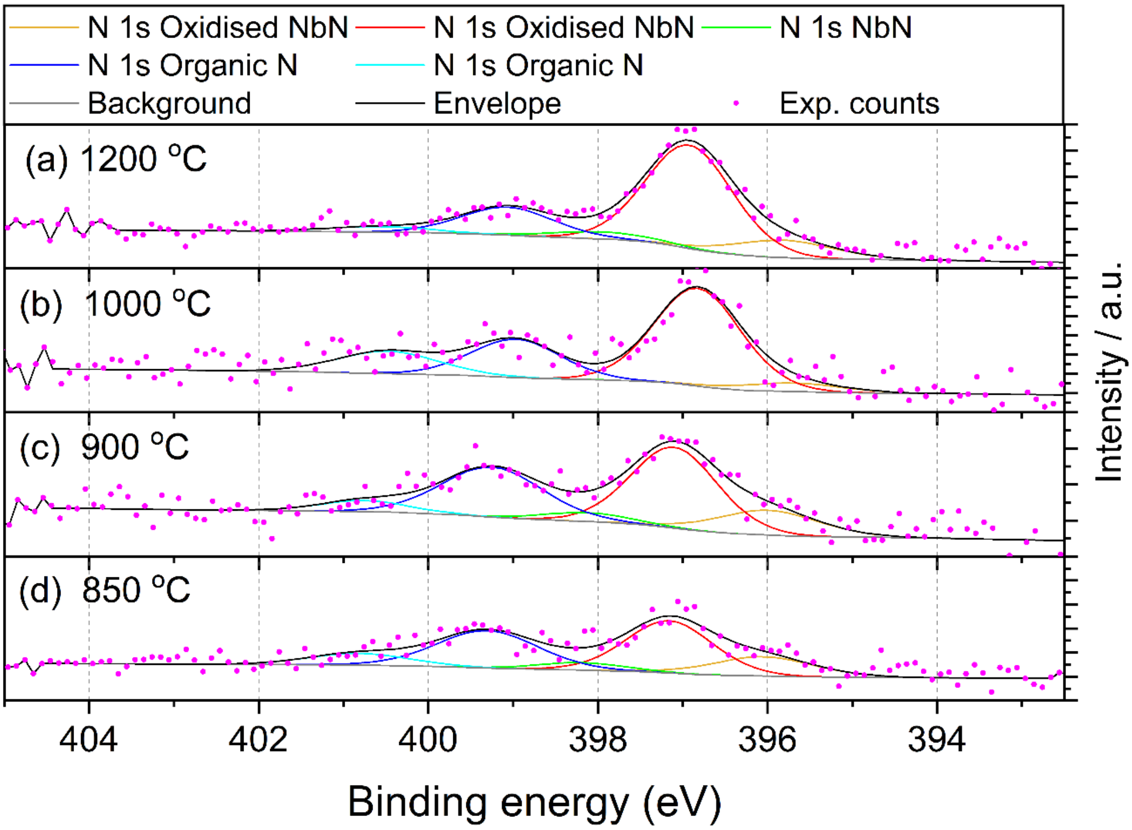

Figure S4: XPS analysis of Nb(C,N) materials in the N 1s region for samples prepared using various synthesis temperatures at 4 hours.

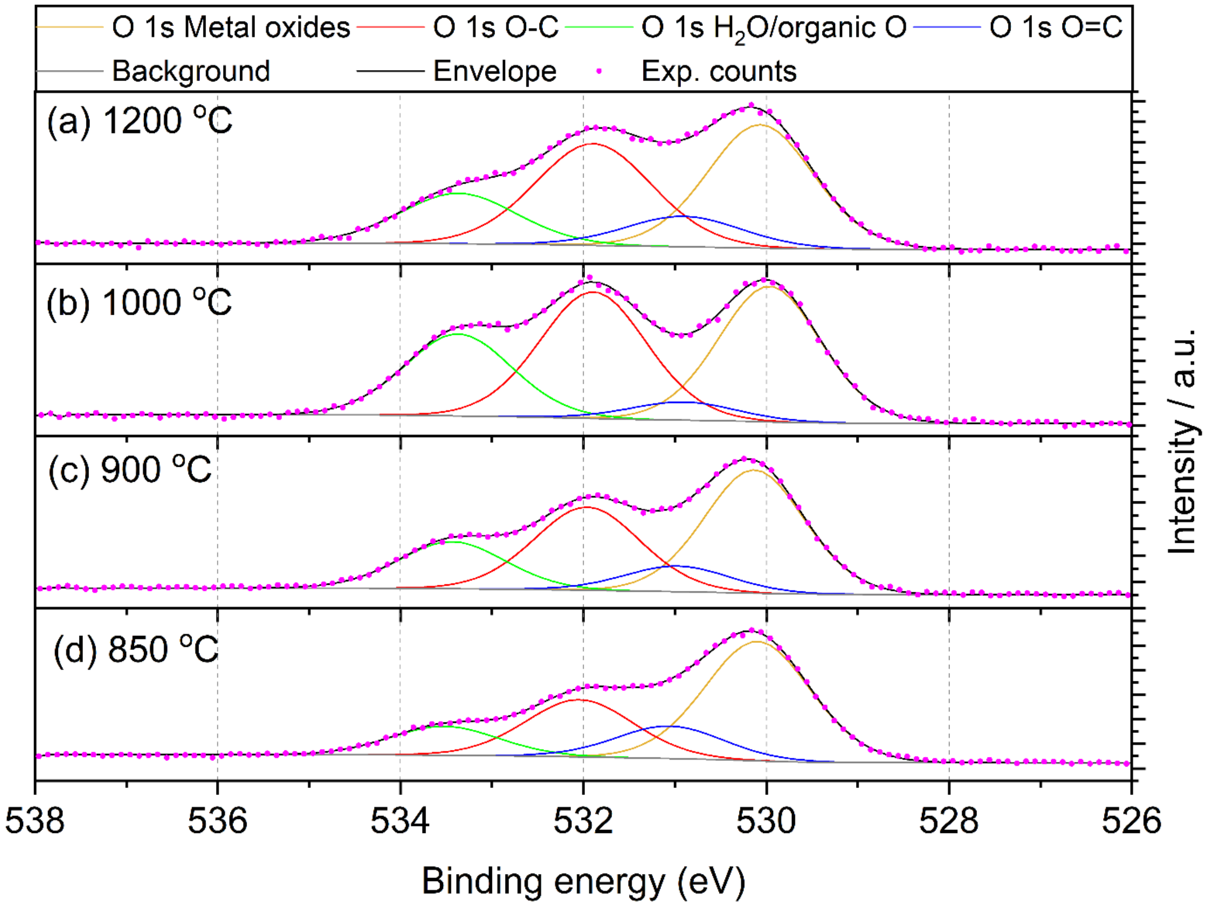

Figure S5: XPS analysis of Nb(C,N) materials in the O 1s region for samples prepared using various synthesis temperatures at 4 hours.

Table S1: Analysis of XPS of Nb(C,N) materials prepared at different temperatures

Synthesis temperature / °C	Nb 3d			C 1s			N 1s			O 1s		
	Binding energy /eV	% of region	Assignment	Binding energy /eV	% of region	Assignment	Binding energy /eV	% of region	Assignment	Binding energy /eV	% of region	Assignment
850	203.62	13.3	Nb $3d_{5/2}$ NbN/NbC	282.39	2.9	NbC	396.04	16.1	Oxidised NbN	530.1	49.6	Metal oxides
	206.39	12.9	Nb $3d_{3/2}$ NbN/NbC	284.8	73.5	C-C/C-H	397.16	37.3	Oxidised NbN	532.04	24.3	O-C
	205.1	1.2	Nb $3d_{5/2}$ NbO_2/ Oxidised NbN	286.41	13.8	C-O	398.19	5.4	NbN	533.52	12.4	H_2O/Organic
	207.82	1.2	Nb $3d_{3/2}$ NbO_2/ Oxidised NbN	287.8	0.1	C=O	399.31	31.3	Organic N	531.06	13.8	O=C /oxidised NbN
	207.04	36.3	Nb $3d_{5/2}$ Nb_2O_5	288.86	9.8	O=C-O	400.82	9.9	Organic N			
	209.76	35.1	Nb $3d_{3/2}$ - Nb_2O_5									

900	203.51	15.4	Nb $3d_{5/2}$ NbN/NbC	282.35	3.2	NbC	395.99	15.3	Oxidised NbN	530.13	42.7	Metal oxides
	206.28	14.9	Nb $3d_{3/2}$ NbN/NbC	284.8	73.7	C-C/C-H	397.1	43.0	Oxidised NbN	531.96	30.6	O-C
	205.22	1.3	Nb $3d_{5/2}$ NbO_2/ Oxidised NbN	286.41	12.3	C-O	398.13	4.7	NbN	533.44	17.3	H_2O/Organic
	207.94	1.2	Nb $3d_{3/2}$ NbO_2/ Oxidised NbN	287.8	0.3	C=O	399.25	30.9	Organic N	530.98	9.4	O=C /oxidised NbN
	207.07	34.1	Nb $3d_{5/2}$ Nb_2O_5	288.79	10.5	O=C-O	400.76	6.1	Organic N			
	209.79	33.0	Nb $3d_{3/2}$ - Nb_2O_5									
1000	203.36	23.8	Nb $3d_{5/2}$ NbN/NbC	282.16	4.8	NbC	395.71	5.7	Oxidised NbN	529.97	36.3	Metal oxides
	206.13	23.0	Nb $3d_{3/2}$ NbN/NbC	284.8	72.79	C-C/C-H	396.82	56.3	Oxidised NbN	531.89	35.5	O-C
	205.37	2.4	Nb $3d_{5/2}$ NbO_2/	286.42	11.9	C-O	397.85	0.1	NbN	533.37	23.1	H_2O/Organic

			Oxidised NbN									
	208.09	2.3	Nb $3d_{3/2}$ NbO_2/ Oxidised NbN	287.8	0.2	C=O	398.97	22.8	Organic N	530.91	5.1	O=C /oxidised NbN
	206.88	24.6	Nb $3d_{5/2}$ Nb_2O_5	288.79	10.4	O=C-O	400.48	15.2	Organic N			
	209.6	23.8	Nb $3d_{3/2}$ - Nb_2O_5									
1200	203.47	26.0	Nb $3d_{5/2}$ NbN/NbC	282.27	8.0	NbC	395.81	12.0	Oxidised NbN	530.07	38.1	Metal oxides
	206.24	25.2	Nb $3d_{3/2}$ NbN/NbC	284.8	71.6	C-C/C-H	396.92	61.4	Oxidised NbN	531.89	34.5	O-C
	205.11	3.0	Nb $3d_{5/2}$ NbO_2/ Oxidised NbN	286.41	11.5	C-O	397.95	4.9	NbN	533.37	17.1	H_2O/Organic
	207.83	2.9	Nb $3d_{3/2}$ NbO_2/ Oxidised NbN	287.8	0.4	C=O	399.07	17.8	Organic N	530.91	10.3	O=C

	207.01	21.9	Nb $3d_{5/2}$ Nb_2O_5	288.79	8.6	O=C-O	400.58	4.0	Organic N			
	209.73	21.2	Nb $3d_{3/2}$ - Nb_2O_5									
